# News and Miscellany

**Published:** 1905-05

**Authors:** 


### News and Miscellany.

---

Wanted—A good location somewhere in West Texas; will buy, exchange, or sell. Am in a good South Texas town of 1200, rich country, good practice; only one other doctor. Want to change on account of health. Would buy out a drug store in a good town. Address, Dr. Eugene, care TEXAS MEDICAL JOURNAL, Austin, Texas.

RARE OPPORTUNITY for the right kind of a doctor: A competent physician with good references can procure a partnership with a well-established physician in large practice in one of the best country site towns in Texas by purchasing an interest in office and fixtures, which are new and complete. Must be energetic and willing to work, and educated "up to date." Adress, "Doctor," care TEXAS MEDICAL JOURNAL, Austin, Texas.

THE meeting of the American Medico-Psychological Association at San Antonio, April 18, 19, 20, will have a write-up in my June number, with the addresses of welcome.

RESIDENT PHYSICIAN WANTED.—A thoroughly, all-round, competent physician is wanted for institutional work. Must be a first-class diagnostician, competent to make all kinds of microscopical examinations, urine analysis, etc. State whether you have own microscope and apparatus. Must furnish with application, gilt-edged recommendations and references. State salary desired in beginning. Address, with stamp, and in own handwriting, Institution, care of TEXAS MEDICAL JOURNAL, Austin, Texas.

THE half-tones in the Houston write-up are ex-presidents of the Texas State Medical Association, with the date of their service.

